# Intensive care services in Hungary 2000 to 2010: an analysis of bed numbers, occupancy rates, case mix and economics

**DOI:** 10.1186/cc11116

**Published:** 2012-03-20

**Authors:** A Csomos, B Fulesdi, M Gresz

**Affiliations:** 1 Semmelweis University, Budapest, Hungary; 2 University of Debrecen, Hungary; 3 National Institute for Quality and Organisational Development in Healthcare, Budapest, Hungary

## Introduction

The purpose of this study is to describe the changes in pattern of intensive care (ICU) use over a 10-year period in Hungary. We attempt to analyze national data in order to improve resource use.

## Methods

A retrospective analysis of national data provided by the hospitals for reimbursement of care to the National Healthcare Fund of Hungary between 2000 and 2010.

## Results

The total number of active hospital beds decreased by 33.4% (from 65,532 to 44,300); however, the number of ICU beds increased by 9.8% (from 1,189 to 1,306) between 2000 and 2010. As a result, the percentage of ICU beds to hospital beds increased from 1.89% in 2000 to 2.95% in 2010. The ICU bed occupancy rate ranged between 58.43% and 63.78%; it showed no correlation with the case mix index (*r*^2 ^= 0.2799). The number of ventilator days increased from 28.9% to 66.1%; it showed good correlation with the case mix index (*r*^2 ^= 0.9125). Analysing 2010 data, we found significantly lower mortality in level III units (30 ± 18%) compared to level II (51 ± 20%) and level I (56 ± 19%) care (*P *= 0.001 and 0.003), without significant differences in case mix index (Table 1). The mean ICU bed occupancy rate was 59.5% (SD ±12%), and length of hospital stay was 12.3 (SD ±3.0) in 2010. Geographic distribution of ICU beds per 100,000 population ranged between 7.3 and 27.4 (nationwide 12.9/100,000); it showed no correlation with regional gross domestic product values (*r*^2 ^= 0.4593).

**Table 1:** Distribution of intensive care services in 2010

National data, 2010	Total number of units	Total number of beds	Case mix index (mean ± SD)	*P *value
University hospitals (level III)	10	412	7.67 (± 4.06)	0.204
County hospitals (level II)	30	584	8.08 (± 2.89)	0.376
City hospitals (level I)	39	280	6.05 (± 1.97)	0.093

## Conclusion

Our data suggest that intensive care beds are not utilized; a progressive level of care does not function and also there are unnecessary regional differences in intensive care provision in Hungary.

